# The Effect of a Single Bout of Continuous Aerobic Exercise on Glucose, Insulin and Glucagon Concentrations Compared to Resting Conditions in Healthy Adults: A Systematic Review, Meta-Analysis and Meta-Regression

**DOI:** 10.1007/s40279-021-01473-2

**Published:** 2021-04-27

**Authors:** James Frampton, Benjamin Cobbold, Mikhail Nozdrin, Htet T. H. Oo, Holly Wilson, Kevin G. Murphy, Gary Frost, Edward S. Chambers

**Affiliations:** 1 Section for Nutrition Research, Department of Metabolism, Digestion and Reproduction, Faculty of Medicine, Imperial College London, London, W12 0NN UK; 2 Section of Endocrinology and Investigative Medicine, Department of Metabolism, Digestion and Reproduction, Faculty of Medicine, Imperial College London, London, W12 0NN UK

## Abstract

**Background:**

Elevated glucose and insulin levels are major risk factors in the development of cardiometabolic disease. Aerobic exercise is widely recommended to improve glycaemic control, yet its acute effect on glycaemia and glucoregulatory hormones has not been systematically reviewed and analysed in healthy adults.

**Objective:**

To determine the effect of a single bout of continuous aerobic exercise on circulating glucose, insulin, and glucagon concentrations in healthy adults.

**Methods:**

CENTRAL, CINAHL, Embase, Global Health, HMIC, Medline, PubMed, PsycINFO, ScienceDirect, Scopus and Web of Science databases were searched from inception to May 2020. Papers were included if they reported a randomised, crossover study measuring glucose and/or insulin and/or glucagon concentrations before and immediately after a single bout of continuous aerobic exercise (≥ 30 min) compared to a time-matched, resting control arm in healthy adults. The risk of bias and quality of evidence were assessed using the Cochrane Risk of Bias Tool and GRADE approach, respectively. Random-effects meta-analyses were performed for glucose, insulin, and glucagon. Sub-group meta-analyses and meta-regression were performed for categorical (metabolic state [postprandial or fasted], exercise mode [cycle ergometer or treadmill]) and continuous (age, body mass index, % males, maximal aerobic capacity, exercise duration, exercise intensity) covariates, respectively.

**Results:**

42 papers (51 studies) were considered eligible: glucose (45 studies, 391 participants), insulin (38 studies, 377 participants) and glucagon (5 studies, 47 participants). Acute aerobic exercise had no significant effect on glucose concentrations (mean difference: − 0.05 mmol/L; 95% CI, − 0.22 to 0.13 mmol/L; *P* = 0.589; *I*^2^: 91.08%, large heterogeneity; moderate-quality evidence). Acute aerobic exercise significantly decreased insulin concentrations (mean difference: − 18.07 pmol/L; 95% CI, − 30.47 to − 5.66 pmol/L; *P* = 0.004; *I*^2^: 95.39%, large heterogeneity; moderate-quality evidence) and significantly increased glucagon concentrations (mean difference: 24.60 ng/L; 95% CI, 16.25 to 32.95 ng/L; *P* < 0.001; *I*^2^: 79.36%, large heterogeneity; moderate-quality evidence). Sub-group meta-analyses identified that metabolic state modified glucose and insulin responses, in which aerobic exercise significantly decreased glucose (mean difference: − 0.27 mmol/L; 95% CI, − 0.55 to − 0.00 mmol/L; *P* = 0.049; *I*^2^: 89.72%, large heterogeneity) and insulin (mean difference: − 42.63 pmol/L; 95% CI, − 66.18 to − 19.09 pmol/L; *P* < 0.001; *I*^2^: 81.29%, large heterogeneity) concentrations in the postprandial but not fasted state. Meta-regression revealed that the glucose concentrations were also moderated by exercise duration and maximal aerobic capacity.

**Conclusions:**

Acute aerobic exercise performed in the postprandial state decreases glucose and insulin concentrations in healthy adults. Acute aerobic exercise also increases glucagon concentrations irrespective of metabolic state. Therefore, aerobic exercise undertaken in the postprandial state is an effective strategy to improve acute glycaemic control in healthy adults, supporting the role of aerobic exercise in reducing cardiometabolic disease incidence.

**PROSPERO registration number:**

CRD42020191345.

**Supplementary Information:**

The online version contains supplementary material available at 10.1007/s40279-021-01473-2.

## Key Points

**Table:** 

A single bout of continuous aerobic exercise significantly decreases glucose concentrations relative to resting conditions in healthy adults when performed in the postprandial state, but not when performed in the fasted state. Changes in glucose concentrations during aerobic exercise are moderated by exercise duration and maximal aerobic capacity.
A single bout of continuous aerobic exercise decreases insulin concentrations relative to resting conditions in healthy adults when performed in the postprandial state, but not when performed in fasted state.
A single bout of continuous aerobic exercise increases glucagon concentrations relative to resting conditions in healthy adults irrespective of metabolic state.

## Introduction

Impaired glycaemic control is a major risk factor in the development of cardiometabolic disease, including type 2 diabetes. Elevated glycated haemoglobin (HbA1c), used as a marker of cumulative glycaemic exposure, independently predicts cardiovascular disease incidence in persons without diabetes [1, 2]. Similarly, postprandial hyperglycaemia and hyperinsulinemia following a standardised glucose bolus predict type 2 diabetes risk in non-diabetic individuals [3, 4]. Pharmaceutical interventions targeting long-term [5] and postprandial [6] glycaemic control in type 2 diabetics prevent macrovascular disease progression. Interventions that improve blood glucose control in non-diabetics may also provide similar benefits with regards to cardiometabolic disease risk and development.

Increasing exercise activity is regarded as an effective strategy for improving glycaemic control [7]. Consequently, engaging in aerobic exercise (e.g. walking, cycling, running) for at least 30 min, five days per week is recommended by various health organisations [8, 9]. Alongside the physiological adaptations induced by aerobic exercise training that can affect glycaemic control [10], blood glucose concentrations are also acutely modulated by aerobic exercise. Blood glucose concentrations are primarily controlled by the pancreatic counterregulatory hormones insulin and glucagon. Circulating levels of both insulin and glucagon can be modified by performing a single bout of exercise [11, 12]. Exercise can also increase glucose uptake independent of insulin action, an effect mediated via increased glucose delivery, transport, and oxidation, and triggered by the metabolic and mechanical stress induced by exercise [13].

There is, however, conflicting evidence regarding the direction and magnitude of changes in glucose, insulin and glucagon concentrations in response to acute aerobic exercise [14–17]. These discrepancies may be explained by small sample sizes, participant (e.g. age, sex) and/or intervention characteristics (e.g. exercise mode, metabolic state). Furthermore, studies have been conducted in both untrained individuals with obesity [18] and elite endurance athletes [19], in whom maximal aerobic capacity likely influences the glycaemic response to acute aerobic exercise. Thus far, meta-analyses investigating the effects of acute aerobic exercise on glycaemic parameters have been limited to individuals with type 1 and 2 diabetes [20–22]. Despite these studies reporting a positive effect of aerobic exercise on acute glycaemic control, these results cannot readily be applied to healthy individuals due to underlying differences in physiology between these two populations [23–25]. This may have important implications for preventing the development of cardiometabolic disease in currently healthy populations. The impact of acute aerobic exercise on glucose, insulin, and glucagon concentrations in healthy individuals, and the influence of potential moderators, is thus currently unknown.

We, therefore, conducted a systematic review and meta-analysis to quantify the glucose, insulin, and glucagon response to a single bout of continuous aerobic exercise relative to resting conditions in healthy adults. Furthermore, we aimed to investigate the role of participant and intervention characteristics on these outcomes using sub-group meta-analyses and meta-regression. The findings from this paper will help to provide a better understanding of the changes in glucose, insulin, and glucagon concentrations with acute aerobic exercise, identify experimental moderators of these responses, and further our understanding of the influence of aerobic exercise on glycaemic control in healthy individuals.

## Methods

### Registration

This Review and Meta-analysis was registered at PROSPERO (registration number: CRD42020191345). PRISMA guidelines were followed throughout the preparation of this manuscript [26].

### Eligibility

#### Inclusion Criteria

To be included in this review and meta-analysis, studies needed to have been a randomised, crossover study measuring glucose and/or insulin and/or glucagon concentrations in plasma or serum before and immediately after (± 5 min exercise cessation) a single bout of continuous aerobic exercise. These two timepoints were selected to evaluate the immediate effect of aerobic exercise on glucose, insulin, and glucagon concentrations that may be lost if investigating a longer time period. The duration of the exercise must have been greater or equal to 30 min and have been performed at a fixed intensity on a treadmill or cycle ergometer. Using a treadmill or cycle ergometer allows exercise intensity to be tightly controlled, guarantees compliance with the protocol relative to self-paced exercise, ensures relative intensity is consistent across participants, and thus permits comparisons within and between studies. A time-matched, resting control arm had to have been performed to negate the effects of time on outcomes, a problem inherent to single-trial studies only comparing pre-and post-exercise concentrations. Consequently, resting and exercise arms had to have been energy-matched (participants in both arms had to have consumed the same meal at the same timepoint). Participants were required to be adults (≥ 18 years) of any body mass index (BMI) value or fitness level.

#### Exclusion Criteria

Studies which were not written in the English language, not published in peer-reviewed journals or included a clamp and/or infusion procedure prior to and/or during the exercise period were excluded. Participants that were pregnant, smoking, currently taking medication that might have interfered with glucose, insulin or glucagon concentrations, had impaired glucose metabolism, or had a history of chronic disease, including type 1 and type 2 diabetes, were also excluded. These exclusion criteria were chosen to prevent self-reported participant characteristics or health conditions from confounding the glucose, insulin, and/or glucagon response to exercise.

Healthy adults were defined as participants that met our inclusion and exclusion criteria. When glucose, insulin and/or glucagon data were not reported in the text (but methods stated measurements had been taken), methodology and/or participant characteristics were not described sufficiently to determine study eligibility, or data were displayed inadequately (e.g. clustering of data points, overlapping of error bars, collating sub-groups), corresponding authors were contacted. If the author did not respond, or could not provide the required data, the study was excluded.

### Database Search

CENTRAL, CINAHL, Embase, Global Health, HMIC, Medline, PubMed, PsycINFO, ScienceDirect, Scopus and Web of Science databases were searched from inception to May 2020. Searches were undertaken between March 2020 and May 2020 using the following concepts and search terms (parentheses): 1. Intervention (‘exercise’, ‘run’, ‘running’, ‘cycle’, ‘cycling’, ‘walk’, ‘walking’), 2. Comparator (‘rest’, ‘resting’, ‘control’, ‘ctrl’, ‘no exercise’, ‘sedentary’), 3. Outcomes (‘glucose’, ‘insulin’, ‘glucagon’), and 4. Study Design (‘crossover’, ‘cross-over’, ‘counterbalanced’). These were then joined (1 AND 2 AND 3 AND 4) to provide the final set of search results. No limits were used during any database search. Full details of the search strategy are provided in Electronic Supplementary Material Appendix S1.

Database results were imported into Covidence systematic review software (Veritas Health Innovation, Australia). Titles and abstracts were independently reviewed by all authors and classified as ‘yes’, ‘no’ or ‘maybe’. Papers classified as ‘yes’ or ‘maybe’ proceeded to the full-text screening stage. Full-text papers were then classified as ‘yes’ or ‘no’ independently by two authors (J.F. and E.S.C), with those classified as ‘yes’ proceeding to the data extraction stage. Any disagreements in paper classification were examined by all authors before coming to an agreement regarding the eligibility of the paper.

### Data Extraction

Data were extracted by a single author (J.F.) into an electronic spreadsheet (Excel 2016, Microsoft Corporation, USA) under the following columns: author; year of publication; sample size; participant characteristics; intervention characteristics; pre- and post-exercise concentrations of glucose and/or insulin and/or glucagon concentrations; and corresponding concentrations of glucose and/or insulin and/or glucagon concentrations in the resting control arm. WebPlotDigitizer Version 4.2 (Ankit Rohatgi, USA) was used to extract data from articles that only presented data in graphical form. If not all error bars were presented, homoscedasticity was assumed and the variance from the timepoint within the same experimental arm was imputed. All extracted data were checked for accuracy by a second author (E.S.C).

Following data extraction, glucose, insulin and glucagon values were converted to SI units (glucose: mmol/L; insulin: pmol/L; glucagon: ng/L). If standard errors or 95% confidence intervals were provided, these were converted to a standard deviation. For each outcome, change scores for exercise and resting arms were calculated by subtracting pre-exercise concentrations from post-exercise concentrations. Mean differences (MDs) between resting and exercise arms were then calculated by subtracting the resting change score from the exercise change score. A positive MD represented an increase in the outcome with exercise, whereas a negative MD represented a decrease with exercise. When within-participant correlation coefficients were not available, a correlation coefficient of 0.5 was assumed to calculate variance and standard error [27]. Sensitivity analyses were performed using correlation coefficients of 0.3, 0.7 and 0.9 to assess the robustness of findings to this assumption.

Studies which reported participants undertaking multiple exercise interventions but only one resting arm were combined into a single change score [28]. Exercise intervention characteristics (duration and intensity) were then averaged. Studies which did not report exercise intensity relative to maximal aerobic capacity ($\dot{V}$O_2_ max) were converted using equations reported previously [29, 30].

### Risk of Bias Assessment

Risk of bias was assessed using the Revised Cochrane Risk of Bias Tool for Randomized trials (RoB 2.0) with additional considerations for cross-over trials. These additional considerations include carryover effects, period effects, and concerns that trials may report only analyses based on the first period. The risk of bias was assessed using the following domains: bias arising from the randomization process; bias due to deviations from intended intervention; bias due to missing outcome data; bias in the measurement of the outcome; and bias in the selection of the reported result. No studies were excluded based on the risk of bias assessment.

### Meta-Analysis Procedures

Data were entered into Stata 16 (StataCorp, USA) for analysis. Data included: participant characteristics (metabolic state [postprandial or fasted], sample size, % males, age, BMI, $\dot{V}$O_2_ max), exercise characteristics (mode [cycle ergometer or treadmill], duration, intensity), mean difference and corresponding standard error. Postprandial exercise was defined as exercise performed within 6 h of meal ingestion. Fasted exercise was defined as the exercise performed 6 h after last meal ingestion.

Simple effect sizes for each outcome were calculated using a random-effects model and with the Sidik–Jonkman approach being employed [31]. All simple effect sizes were presented as (unstandardised) MDs and using SI units to facilitate interpretability of results. A random-effects model was chosen over a fixed-effects model to account for differences in participant characteristics and methodology between studies [32]. Heterogeneity was assessed using the chi-squared (*Q*) and *I*^2^ statistic. A *Q* value above the degrees of freedom (*df*) for the estimate and an *I*^2^ statistic > 50% indicated large heterogeneity between studies. To investigate the influence of participant characteristics and methodology on MDs, sub-group meta-analyses (categorical covariates) and random-effects meta-regression (continuous covariates) were performed. For the random-effects meta-regression, a positive coefficient indicated that an increase in the covariate was associated with an increase in glucose/insulin/glucagon concentrations with exercise. A negative coefficient indicated that an increase in the covariate was associated with a decrease in glucose/insulin/glucagon concentrations with exercise. Metabolic state and exercise mode were analysed as categorical covariates; % males, age, BMI, $\dot{V}$O_2_ max, exercise duration and exercise intensity were analysed as continuous covariates. Publication bias was assessed using visual inspection of contour-enhanced funnel plots [33] and statistically by Egger’s regression test. Trim and fill analyses were used when publication bias was suspected to explore its impact on MDs. Statistical significance was set at *P* < 0.05 in a *Z* test analysis. *Z* tests were used to examine if MDs were significantly different from zero. Results are displayed as overall MDs with 95% confidence intervals (CI).

### Quality of Evidence Assessment

The quality of evidence was assessed using the strategy recommended by the Grading of Recommendations Assessment Development and Evaluation (GRADE) working group [34]. The quality of evidence was assessed using the following domains: risk of bias; inconsistency; indirectness; imprecision; and publication bias. The estimated effect for each outcome was then classified as very low (true effect is probably markedly different from the estimated effect), low (true effect might be markedly different from the estimated effect), moderate (true effect is probably close to the estimated effect) or high quality (true effect is similar to the estimated effect).

## Results

**Fig. 1:**
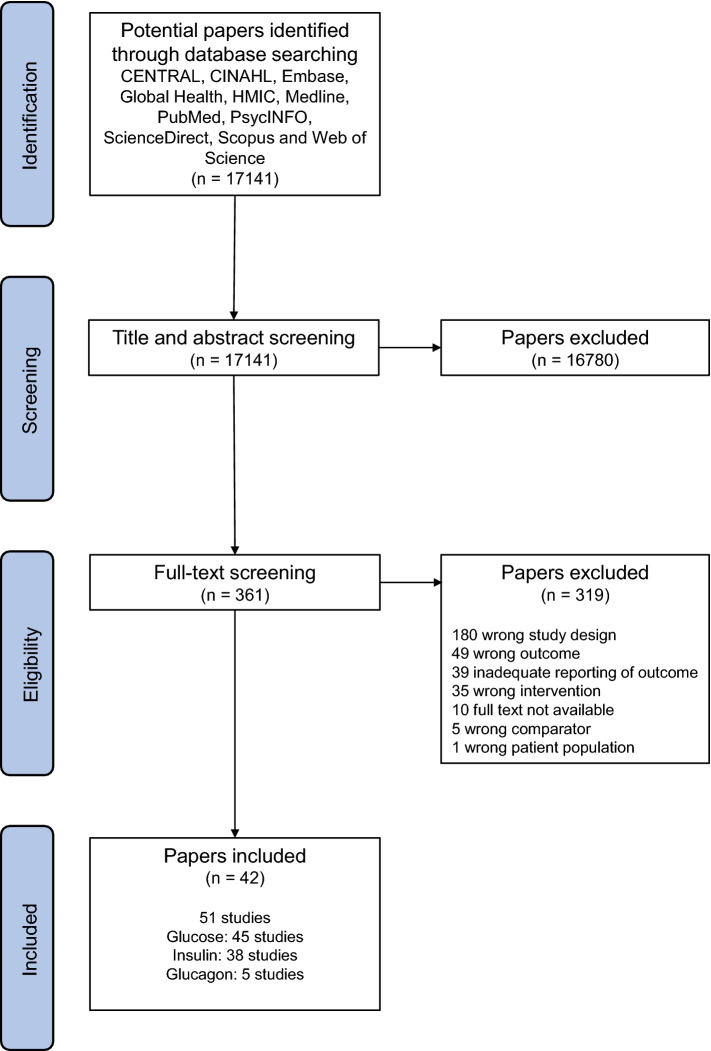
Flow diagram of paper selection

Database searches identified 17,141 potentially eligible papers. Title and abstract screening resulted in the exclusion of 16,780 papers, resulting in 361 papers being assessed for eligibility by full-text inspection. Screening of full texts identified 42 papers which were eligible to be included in the review and meta-analysis. Due to several papers containing multiple studies, a total of 51 separate studies were included in the analysis. Consequently, each outcome comprised the following number of studies and total participants—glucose: 45 studies, 391 participants; insulin: 38 studies, 377 participants; glucagon: 5 studies, 47 participants. This process is summarised in Fig. 1. Details of the included studies are displayed in Table 1.

**Table 1:** Participant characteristics, intervention characteristics and outcome measurements for all included studies

Study	Participant characteristics	Intervention characteristics	Glucose (mmol/L)	Insulin (pmol/L)	Glucagon (ng/L)
Bahr et al. [35]	12 males; fasted Age: 23.0 ± 1.7 $\dot{V}$O_2_ max: 52 ± 3.6	Cycle ergometer 63 min 62% $\dot{V}$O_2_ max	CON: 0.04 ± 0.44 EX: − 0.73 ± 0.53	NM	NM
Balaguera-Cortes et al. [36]	10 males; fasted Age: 21.3 ± 1.4; BMI: 23.7 ± 2.0 $\dot{V}$O_2_ max: 58.1 ± 7.3	Treadmill 45 min 70% $\dot{V}$O_2_ max	CON: 0.00 ± 0.46 EX: 0.20 ± 0.44	CON: − 10.66 ± 24.67 EX: 6.71 ± 19.64	NM
Bergfors et al. [37]	10 males; fasted Age: 26.7 ± 6.6; BMI: 23.1 ± 2.2	Cycle ergometer 37 min 60% $\dot{V}$O_2_ max	CON: − 0.10 ± 0.32 EX: 0.00 ± 0.55	CON: − 4.20 ± 16.85 EX: − 19.80 ± 14.32	NM
Broom et al. [38]^a^	9 males; fasted Age: 21.4 ± 1.7; BMI: 24.5 ± 2.4 $\dot{V}$O_2_ max: 58 ± 6	Treadmill 55 min 52% $\dot{V}$O_2_ max	CON: −0.19 ± 0.87 EX: −0.47 ± 0.72	CON: −0.34 ± 36.37 EX: −47.36 ± 62.09	NM
Broom et al.[38]^b^	9 males; fasted Age: 23.2 ± 2.1; BMI: 22.7 ± 1.5 $\dot{V}$O_2_ max: 63 ± 6	Treadmill 68 min^c^ 70% $\dot{V}$O_2_ max	CON: −0.18 ± 0.20 EX: 0.60 ± 0.58	CON: − 5.24 ± 10.37 EX: 13.41 ± 18.53	NM
Burns et al. [15]	9 males; 9 females; fasted Age: 24.8 ± 3.8; BMI: 22.9 ± 2.7 $\dot{V}$O_2_ max: 57.7 ± 7.4	Treadmill 60 min 75% $\dot{V}$O_2_ max	CON: − 0.09 ± 0.67 EX: 1.37 ± 1.51	CON: − 3.46 ± 43.25 EX: − 20.06 ± 55.44	NM
Charlot et al. [39]	9 males; postprandial Age: 21.9 ± 1.8; BMI: 22.7 ± 1.6 $\dot{V}$O_2_ max: 49 ± 9	Cycle ergometer 75 min 70% $\dot{V}$O_2_ max	CON: − 0.26 ± 0.59 EX: − 0.95 ± 0.81	NM	NM
Clegg et al. [40]	8 males; fasted Age: 22.9 ± 2.8	Cycle ergometer 60 min 35% $\dot{V}$O_2_ max^d^	CON: − 0.16 ± 0.34 EX: − 0.36 ± 0.28	NM	NM
Douglas et al. [41]^a^	11 males, 11 females; fasted Age: 37.5 ± 15.2; BMI: 22.4 ± 1.5 $\dot{V}$O_2_ max: 43.6 ± 12.2	Treadmill 60 min 60% $\dot{V}$O_2_ max	CON: − 0.18 ± 0.19 EX: 0.27 ± 0.64	CON: − 4.66 ± 7.98 EX: − 1.68 ± 13.01	NM
Douglas et al. [41]^b^	14 males, 11 females; fasted Age: 45.0 ± 12.4; BMI: 29.2 ± 2.9 $\dot{V}$O_2_ max: 34.7 ± 8.9	Treadmill 60 min 60% $\dot{V}$O_2_ max	CON: −0.16 ± 0.28 EX: 0.29 ± 0.45	CON: −1.53 ± 12.27 EX: 3.89 ± 21.09	NM
Edinburgh et al. [42]	10 males; fasted Age: 23.0 ± 3.0; BMI: 23.3 ± 1.8 $\dot{V}$O_2_ max: 52.7 ± 8.9	Cycle ergometer 60 min 63% $\dot{V}$O_2_ max	CON: −0.03 ± 0.17 EX: −0.20 ± 0.53	CON: −2.45 ± 3.27 EX: −2.33 ± 7.49	NM
Enevoldsen et al. [14]	6 males; postprandial Age: 25 (23–28)^e^	Cycle ergometer 60 min 50% $\dot{V}$O_2_ max	CON: −1.70 ± 0.93 EX: −2.57 ± 0.71	CON: −145.00 ± 111.48 EX: −220.00 ± 51.32	NM
Ezell et al. [43]^a^	5 females; postprandial Age: 25.6 ± 7.8; BMI: 20.6 ± 2.1 $\dot{V}$O_2_ max: 33.0 ± 7.2	Cycle ergometer 60 min 63% $\dot{V}$O_2_ max	CON: 0.44 ± 0.62 EX: 0.10 ± 0.72	CON: −83.40 ± 125.43 EX: −105.60 ± 72.20	NM
Ezell et al. [43]^b^	5 females; postprandial Age: 26.2 ± 6.3; BMI: 30 ± 6.0 $\dot{V}$O_2_ max: 22.1 ± 6.8	Cycle ergometer 60 min 63% $\dot{V}$O_2_ max	CON: −0.21 ± 0.51 EX: −0.60 ± 0.71	CON: −112.80 ± 81.91 EX: −130.20 ± 115.22	NM
Ezell et al. [43]^f^	5 females; postprandial Age: 22.6 ± 2.3; BMI: 22.7 ± 3.0 $\dot{V}$O_2_ max: 30 ± 6.5	Cycle ergometer 60 min 63% $\dot{V}$O_2_ max	CON: −0.01 ± 0.59 EX: −0.37 ± 0.49	CON: −34.20 ± 17.59 EX: −33.60 ± 27.37	NM
Farah and Gill [44]	10 males; postprandial Age: 28.1 ± 10.7; BMI: 29.0 ± 2.8 $\dot{V}$O_2_ max: 39.1 ± 5.4	Treadmill 60 min 50% $\dot{V}$O_2_ max	CON: −1.12 ± 1.03 EX: −0.12 ± 0.68	CON: −198.60 ± 199.56 EX: −231.24 ± 100.78	NM
Gonzalez et al. [45]^a^	11 males; postprandial Age: 23.2 ± 4.3; BMI: 24.5 ± 2.0 $\dot{V}$O_2_ max: 53.1 ± 5.5	Treadmill 59 min 61% $\dot{V}$O_2_ max	CON: 0.57 ± 0.29 EX: 0.60 ± 0.81	CON: −70.30 ± 63.69 EX: −158.64 ± 100.94	NM
Gonzalez et al. [45]^b^	11 males; fasted Age: 23.2 ± 4.3; BMI: 24.5 ± 2.0 $\dot{V}$O_2_ max: 53.1 ± 5.5	Treadmill 59 min 61% $\dot{V}$O_2_ max	CON: 0.07 ± 0.19 EX: 0.52 ± 0.26	CON: −9.04 ± 26.15 EX: −52.82 ± 22.28	NM
Goto et al. [46]	9 males; fasted Age: 24.0 ± 2.1; BMI: 22.1 ± 1.8	Cycle 30 min 60% $\dot{V}$O_2_ max	CON: −0.08 ± 0.47 EX: 0.38 ± 0.44	NM	NM
Hagobian et al. [47]^a^	11 males; fasted Age: 22 ± 2; BMI: 26 ± 4 $\dot{V}$O_2_ max: 42.9 ± 6.5	Cycle ergometer 82 min 70% $\dot{V}$O_2_ max	NM	CON: −26.40 ± 32.67 EX: −30.60 ± 31.63	NM
Hagobian et al. [47]^b^	10 females; fasted Age: 21 ± 2; BMI: 24 ± 2 $\dot{V}$O_2_ max: 39.9 ± 5.5	Cycle ergometer 84 min 70% $\dot{V}$O_2_ max	NM	CON: −15.00 ± 26.09 EX: −24.00 ± 7.87	NM
Hardman and Aldred [48]	6 males, 6 females; postprandial Age: 26.0 ± 5.2; BMI: 23.95 ± 1.6 $\dot{V}$O_2_ max: 48.2 ± 11.9	Treadmill 90 min 40% $\dot{V}$O_2_ max	NM	CON: −11.88 ± 35.62 EX: −61.38 ± 67.22	NM
Højbjerre et al. [49]^a^	8 males; fasted Age: 26.0 ± 2.0; BMI: 22.8 ± 1.4 $\dot{V}$O_2_ max: 57.1 ± 4.2	Cycle ergometer 60 min 55% $\dot{V}$O_2_ max	CON: −0.01 ± 0.21 EX: −0.35 ± 0.52	NM	NM
Højbjerre et al. [49]^b^	8 males; fasted Age: 26.3 ± 2.3; BMI: 28.0 ± 0.8 $\dot{V}$O_2_ max: 54.6 ± 6.2	Cycle ergometer 60 min 55% $\dot{V}$O_2_ max	CON: −0.10 ± 0.13 EX: −0.26 ± 0.39	NM	NM
Isacco et al. [50]^a^	10 females; postprandial Age: 22.9 ± 3.5; BMI: 22.0 ± 3.2 $\dot{V}$O_2_ max: 54.8 ± 5.4	Cycle ergometer 45 min 65% $\dot{V}$O_2_ max	CON: 0.29 ± 0.54 EX: −0.54 ± 1.04	CON: −121.40 ± 143.92 EX: −85.19 ± 103.27	NM
Isacco et al. [50]^b^	11 females; postprandial Age: 21.2 ± 2.0; BMI: 22.6 ± 2.0 $\dot{V}$O_2_ max: 50.4 ± 7.6	Cycle ergometer 45 min 65% $\dot{V}$O_2_ max	CON: 0.01 ± 0.45 EX: −0.16 ± 0.90	CON: −29.56 ± 59.64 EX: −55.99 ± 48.94	NM
King et al. [51]	14 males; fasted Age: 21.9 ± 1.9; BMI: 23.4 ± 2.2 $\dot{V}$O_2_ max: 55.9 ± 6.7	Treadmill 60 min 45% $\dot{V}$O_2_ max	CON: 0.01 ± 0.60 EX: 0.03 ± 0.56	CON: 5.42 ± 28.47 EX: −9.78 ± 23.87	NM
Knudsen et al. [25]	7 Males; fasted Age: 57.0 ± 3.7; BMI: 26.8 ± 5.0 $\dot{V}$O_2_ max: 36.4 ± 5.8	Cycle ergometer 60 min 55% $\dot{V}$O_2_ max^d^	NM	CON: −2.81 ± 6.18 EX: −4.10 ± 4.61	NM
Larsen et al. [52]	12 males; fasted Age: 48.0 ± 5.0; BMI: 29.9 ± 1.9 $\dot{V}$O_2_ max: 31.0 ± 8.0	Cycle ergometer 50 min 78% $\dot{V}$O_2_ max	NM	CON: −9.64 ± 12.05 EX: −20.56 ± 12.05	CON: −6.50 ± 5.25 EX: 17.44 ± 8.58
Lee et al. [53]	12 males; fasted Age: 36.9 ± 7.6 $\dot{V}$O_2_ max: 26.3 ± 7.5	Treadmill 45 min 60% $\dot{V}$O_2_ max	CON: −0.26 ± 0.46 EX: 0.01 ± 0.76	NM	NM
Marion-Latard et al. [54]	6 males; postprandial Age: 30.7 ± 6.9; BMI: 31.8 ± 2.5 $\dot{V}$O_2_ max: 33.2 ± 4.7	Cycle ergometer 60 min 50% $\dot{V}$O_2_ max	CON: 0.22 ± 0.49 EX: −0.20 ± 0.92	CON: −30.48 ± 33.74 EX: −32.22 ± 30.51	NM
Mattin et al. [55]	12 males; fasted Age: 26.0 ± 5.0; BMI: 25.5 ± 3.5 $\dot{V}$O_2_ max: 42.2 ± 6.6	Cycle ergometer 60 min 55% $\dot{V}$O_2_ max^c^	CON: −0.12 ± 0.29 EX: 0.14 ± 0.29	CON: 0.29 ± 24.87 EX: 2.32 ± 30.42	NM
Mc Clean et al. [56]	10 males; postprandial Age: 21.5 ± 2.5; BMI: 23.6 ± 1.6 $\dot{V}$O_2_ max: 58.5 ± 7.1	Treadmill 60 min 35% $\dot{V}$O_2_ max^d^	CON: 0.25 ± 0.38 EX: 0.51 ± 0.34	NM	NM
Morris et al. [57]	6 males; postprandial Age: 30.0 ± 8.0; BMI: 23.1 ± 1.1 $\dot{V}$O_2_ max: 49 ± 7	Cycle ergometer 60 min 50% $\dot{V}$O_2_ max	CON: −0.38 ± 0.88 EX: −0.22 ± 0.78	CON: −4.04 ± 24.47 EX: −10.44 ± 17.86	NM
Numao et al. [58]	8 Males; fasted Age: 24.9 ± 1.7; BMI: 21.9 ± 1.4 $\dot{V}$O_2_ max: 52.8 ± 5.1	Cycle ergometer 60 min 50% $\dot{V}$O_2_ max	CON: −0.10 ± 0.28 EX: −0.50 ± 0.28	CON: −10.90 ± 9.48 EX: −24.30 ± 17.82	NM
Nyhoff et al. [18]	11 females; postprandial Age: 24.3 ± 4.6; BMI: 37.3 ± 7.0 $\dot{V}$O_2_ max: 25.2 ± 4.6	Treadmill 55 min 55% $\dot{V}$O_2_ max	CON: −0.05 ± 0.69 EX: −0.31 ± 0.66	CON: −16.20 ± 172.79 EX: −108.80 ± 126.64	CON: −4.84 ± 4.78 EX: 15.10 ± 4.78
Petridou et al. [59]	11 males; fasted Age: 21.7 ± 2.0; BMI: 22.5 ± 1.6	Cycle ergometer 45 min 40% $\dot{V}$O_2_ max^d^	CON: −0.14 ± 0.70 EX: −0.21 ± 0.70	CON: −13.80 ± 55.41 EX: −4.56 ± 62.56	NM
Rattray and Smee [60]	10 males, 10 females; fasted Age: 25.6 ± 5.4 $\dot{V}$O_2_ max: 49.6 ± 8.1	Cycle ergometer 60 min 60% $\dot{V}$O_2_ max^d^	CON: −0.75 ± 0.68 EX: −0.47 ± 0.96	NM	NM
Ronsen et al. [19]	9 males; postprandial Age: 21–27^e^; $\dot{V}$O_2_ max: 69.1 ± 11.1	Cycle ergometer 65 min 75% $\dot{V}$O_2_ max	NM	CON: −32.17 ± 70.47 EX: −127.08 ± 19.74	NM
Ronsen et al. [61]	9 males; postprandial $\dot{V}$O_2_ max: 69.1 ± 11.1	Cycle ergometer 65 min 75% $\dot{V}$O_2_ max	CON: 0.14 ± 0.57 EX: − 1.27 ± 0.63	NM	NM
Schlierf et al. [62]	12 males; postprandial Age: 25 (21–37)^e^	Cycle ergometer 90 min 40% $\dot{V}$O_2_ max	CON: 0.55 ± 0.61 EX: 0.89 ± 0.62	CON: -10.80 ± 57.47 EX: -68.40 ± 75.65	NM
Shambrook et al. [63]	10 males; postprandial Age: 37.3 ± 7.3; BMI: 29.3 ± 6.5 $\dot{V}$O_2_ max: 33.7 ± 7.4	Cycle ergometer 30 min 42% $\dot{V}$O_2_ max^c^	CON: −0.58 ± 0.73 EX: −1.14 ± 0.64	NM	NM
Shambrook et al. [64]	8 males, 2 females; postprandial Age: 50.0 ± 12.6; BMI: 29.0 ± 5.4 $\dot{V}$O_2_ max: 32.6 ± 6.5	Treadmill 30 min 63% $\dot{V}$O_2_ max^d^	CON: 0.27 ± 0.28 EX: −0.85 ± 0.37	NM	NM
Siopi et al. [17]	14 males; fasted Age: 41.0 ± 7.0; BMI: 28.1 ± 4.2 $\dot{V}$O_2_ max: 37.0 ± 4.1	Treadmill 36 min 40% $\dot{V}$O_2_ max^d^	CON: 0.06 ± 0.55 EX: 0.00 ± 0.40	CON: −18.00 ± 33.41 EX: 0.00 ± 43.27	NM
Stokes et al. [65]	8 males; fasted Age: 22.0 ± 1.0 $\dot{V}$O_2_ max: 53.0 ± 6.0	Cycle ergometer 30 min 70% $\dot{V}$O_2_ max	CON: 0.06 ± 0.33 EX: −0.05 ± 0.38	NM	NM
Tobin et al. [24]	7 males; postprandial Age: 58.0 ± 3.2; BMI: 28.0 ± 2.4 $\dot{V}$O_2_ max: 33.6 ± 6.4	Cycle ergometer 60 min 53 $\dot{V}$O_2_ max	CON: 0.00 ± 0.63 EX: 0.51 ± 0.74	CON: 16.12 ± 76.28 EX: − 12.14 ± 78.69	NM
Ueda et al. [66]	10 males; postprandial Age: 23.4 ± 4.3 BMI: 22.5 ± 1.0 $\dot{V}$O_2_ max: 45.9 ± 8.5	Cycle ergometer 30 min 63% $\dot{V}$O_2_ max^c^	CON: −0.13 ± 0.89 EX: −1.85 ± 1.24	CON: −21.42 ± 71.10 EX: −182.24 ± 55.07	NM
Ueda et al. [16]^a^	7 males; postprandial Age: 22.4 ± 4.2; BMI: 22.4 ± 2.4 $\dot{V}$O_2_ max: 46.6 ± 3.9	Cycle ergometer 60 min 50% $\dot{V}$O_2_ max	CON: − 0.18 ± 0.74 EX: − 0.12 ± 0.56	CON: − 57.72 ± 86.12 EX: − 84.84 ± 101.50	CON: 3.64 ± 61.70 EX: 52.35 ± 62.09
Ueda et al. [16]^b^	7 males; postprandial Age: 22.9 ± 3.4; BMI: 30.0 ± 3.1 $\dot{V}$O_2_ max: 34.0 ± 6.3	Cycle ergometer 60 min 50% $\dot{V}$O_2_ max	CON: −0.16 ± 0.38 EX: 0.09 ± 0.45	CON: −144.72 ± 153.07 EX: −159.30 ± 182.50	CON: 4.77 ± 66.35 EX: 23.56 ± 46.21
Vendelbo et al. [67]	8 males; fasted Age: 25.5 ± 12.2; BMI: 23.8 ± 5.5	Cycle ergometer 60 min 65% $\dot{V}$O_2_max	CON: 0.00 ± 0.31 EX: 0.40 ± 0.56	CON: 1.00 ± 18.55 EX: 11.00 ± 32.62	NM
Willis et al. [68]	10 males; fasted Age: 26.0 ± 2.0; BMI: 25.6 ± 1.7 $\dot{V}$O_2_ max: 49.8 ± 5.3	Treadmill 50 min^c^ 65% $\dot{V}$O_2_ max^c^	CON: −0.01 ± 1.32 EX: 0.94 ± 1.32	CON: 0.06 ± 19.30 EX: 2.33 ± 19.30	CON: −6.48 ± 14.78 EX: 25.32 ± 23.85

Data expressed as mean ± SD; Participant characteristic (units): years (age), BMI (kg/m^2^) and $\dot{V}$O_2_ max (ml/min/kg)

*CON* control arm, *EX* exercise arm, *NM* not measured or data could not be extracted

^a,b,f^After author names denotes sub-studies; ^c^averaged value across two sub-studies; ^d^converted to $\dot{V}$O_2_ max; ^e^only range provided

### Risk of Bias Analysis

A risk of bias summary table is presented in Electronic Supplementary Material Appendix S2.

Most studies measuring glucose (93%) and insulin (97%) concentrations were classified as possessing an unclear risk of bias overall (Electronic Supplementary Material Figure S1a and 1b). All studies measuring glucagon concentrations were classified as having an unclear risk of bias overall (Electronic Supplementary Material Figure S1c).

### Meta-Analysis

#### Glucose

**Fig. 2:**
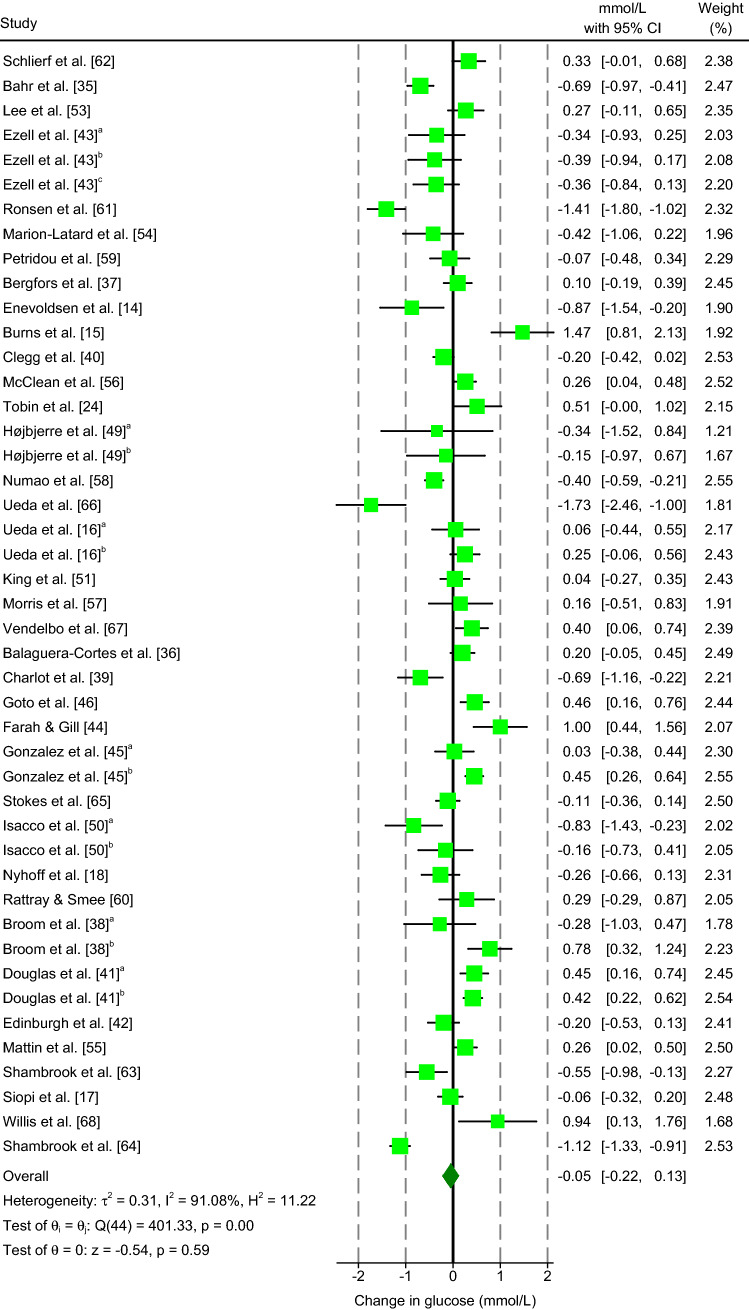
Forest plot of simple effect sizes for studies assessing the effect of a single bout of continuous aerobic exercise on glucose concentrations (mmol/L). Data are presented as mean difference ± 95% CI. Random-effects Sidik–Jonkman model. ^a,b,c^ denotes sub-studies. Ezell et al. [43]^c^ refers to Ezell et al. [43]^f^ in Table 1

The results of the meta-analysis revealed that aerobic exercise non-significantly decreased glucose concentrations compared to resting conditions (MD: −0.05 mmol/L; 95% CI, − 0.22 to 0.13 mmol/L; *P* = 0.589; *n* = 45; Fig. 2). *I*^2^ (91.08%) and *Q* (401.33, *df* = 44, *P* < 0.001) statistics highlighted large heterogeneity between studies.

Sub-group meta-analyses of categorical covariates (metabolic state and exercise mode) highlighted a significant difference in MDs between postprandial and fasted aerobic exercise (*P* = 0.013; Electronic Supplementary Material Figure S2). Postprandial aerobic exercise significantly decreased glucose concentrations (MD: − 0.27 mmol/L; 95% CI, − 0.55 to − 0.00 mmol/L; *P* = 0.049; *n* = 22) and fasted aerobic exercise non-significantly increased glucose concentrations (MD: 0.15 mmol/L; 95% CI, − 0.04 to 0.34 mmol/L; *P* = 0.122; *n* = 23) relative to resting conditions. Sub-group analysis resulted in only a small reduction in the *I*^2^ statistic (postprandial: 89.72%; fasted: 87.75%). A significant difference in MDs between exercise modalities (cycle ergometer vs treadmill) was also observed (*P* = 0.008; Electronic Supplementary Material Figure S3). Exercise performed on a cycle ergometer significantly decreased glucose concentrations (MD: − 0.22 mmol/L; 95% CI, − 0.42 to − 0.03 mmol/L; *P* = 0.026; *n* = 29) and on a treadmill non-significantly increased glucose concentrations (MD: 0.26 mmol/L; 95% CI, − 0.04 to 0.55 mmol/L; *P* = 0.085; *n* = 16) compared to resting conditions. The sub-group analysis resulted in a small decrease in the *I*^2^ statistic for studies using a cycle ergometer (86.18%) but a small increase in those using a treadmill (92.92%).

Random-effects meta-regression identified two significant moderator covariates: $\dot{V}$O_2_ max and exercise duration. Both $\dot{V}$O_2_ max (coefficient: 0.033; 95% CI, 0.001 to 0.064; *P* = 0.045) and exercise duration (coefficient: 0.030; 95% CI, 0.011 to 0.049; *P* = 0.002; Electronic Supplementary Material Appendix S3) showed a positive correlation with glucose concentrations.

Visual inspection of the contour-enhanced funnel plot implied a symmetrical distribution, suggesting no evidence of publication bias (Electronic Supplementary Material Figure S4a). This was confirmed by results from Egger’s regression test (*P* = 0.604).

#### Insulin

**Fig. 3:**
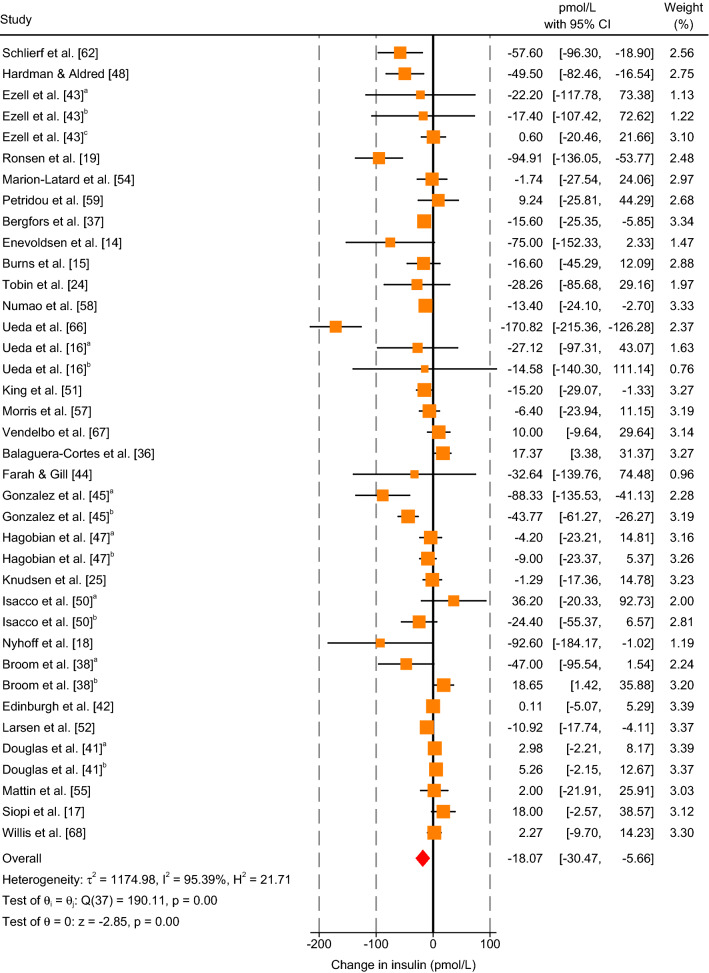
Forest plot of simple effect sizes for studies assessing the effect of a single bout of continuous aerobic exercise on insulin concentrations (pmol/L). Data are presented as mean difference ± 95% CI. Random-effects Sidik–Jonkman model. ^a,b,c^ denotes sub-studies. Ezell et al. [43]^c^ refers to Ezell et al. [43]^f^ in Table 1

The results of the meta-analysis revealed that aerobic exercise significantly decreased insulin concentrations relative to resting conditions (MD: − 18.07 pmol/L; 95% CI, − 30.47 to − 5.66 pmol/L; *P* = 0.004; *n* = 38; Fig. 3). *I*^2^ (95.39%) and *Q* (190.11, *df* = 37, *P* < 0.001) statistics highlighted large heterogeneity among studies.

Sub-group meta-analysis of categorical covariates (metabolic state and exercise mode) highlighted a significant difference in MDs between postprandial and fasted aerobic exercise (*P* = 0.002; Electronic Supplementary Material Figure S5). Postprandial aerobic exercise significantly decreased insulin concentrations (MD: − 42.63 pmol/L; 95% CI, − 66.18 to − 19.09 pmol/L; *P* < 0.001; *n* = 18), whereas fasted aerobic exercise non-significantly decreased insulin concentrations (MD: − 3.40 pmol/L; 95% CI, − 10.74 to 3.94; *P* = 0.370; *n* = 20) compared to resting conditions. Sub-group analysis resulted in only a small reduction in the *I*^2^ statistic (postprandial: 81.29%; fasted: 86.69%). No effect of exercise mode was detected (*P* = 0.726; Electronic Supplementary Material Figure S6). Aerobic exercise performed using a cycle ergometer significantly decreased insulin concentrations (MD: -19.67 pmol/L; 95% CI, −36.39 to −2.95 pmol/L; *P* = 0.021; *n* = 23), whereas using a treadmill non-significantly decreased insulin concentrations (MD: − 15.22 pmol/L; 95% CI, − 33.63 to 3.19 pmol/L; *P* = 0.105; *n* = 15) relative to resting conditions. Sub-group analysis resulted in only a small reduction in the *I*^2^ statistic (cycle ergometer = 95.06%; treadmill = 94.75%). Random-effects meta-regression showed no significant moderator effects of continuous covariates (Electronic Supplementary Material Appendix S3).

Visual inspection of the contour-enhance funnel plot showed a distribution to the left, suggesting publication bias (Electronic Supplementary Material Figure S4b). However, studies appear to be missing from non-significant (dark grey) and significant (light grey and white) regions, indicating that funnel plot asymmetry maybe due to other factors such as heterogeneity. Based on the results of the sub-group meta-analysis showing a significant difference in MDs between postprandial and fasted exercise, separate contour-enhanced funnel plots were generated for each metabolic state (Electronic Supplementary Material Figure S4c). Funnel plots for postprandial and fasted exercise displayed an approximal symmetrical distribution, which was confirmed by Egger’s regression test with metabolic state included as moderator (*P* = 0.404).

#### Glucagon

**Fig. 4:**
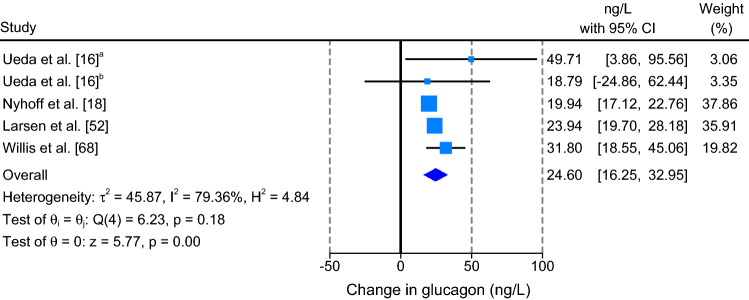
Forest plot of simple effect sizes for studies assessing the effect of a single bout of continuous aerobic exercise on glucagon concentrations (ng/L). Data are presented as mean difference ± 95% CI. Random-effects Sidik–Jonkman model. ^a,b^ denotes sub-studies

The results of the meta-analysis revealed that aerobic exercise significantly increased glucagon concentrations compared to resting conditions (MD: 24.60 ng/L; 95% CI, 16.25 to 32.95 ng/L; *P* < 0.001; *n* = 5; Fig. 4). *I*^2^ (79.36%) and *Q* (6.23, *df *= 4, *P* = 0.183) statistics highlighted large heterogeneity between studies.

Due to the small number of studies reporting glucagon concentrations, sub-group meta-analyses and meta-regression were not performed. Visual inspection of contour-enhanced funnel plots did not suggest large asymmetry and thus no evidence of publication bias (Electronic Supplementary Material Figure S4d). This was confirmed by the results of Egger’s regression test (*P* = 0.357).

#### Sensitivity Analyses

Sensitivity analyses employing within-participant correlation coefficients of 0.3, 0.7 and 0.9 did not affect the significance of the MDs for insulin, glucagon or glucose (Electronic Supplementary Material Appendix S4).

### Quality of Evidence

The effect estimates for insulin, glucagon, and glucose outcomes were all categorised as moderate quality. Insulin, glucagon, and glucose were all downgraded by one level due to inconsistency of results, as the large heterogeneity observed for all three outcomes could not be explained by sub-group analyses or meta-regression. A summary of findings is presented in Table 2.

**Table 2:** Summary of findings for glucose, insulin and glucagon outcomes.

Acute continuous aerobic exercise compared with resting conditions in healthy adults				
Patient or population: healthy adults Setting: laboratory environment Intervention: acute continuous aerobic exercise Comparison: rest				
Outcomes	Relative effect (95% CI)	Number of participants (studies)	Quality of the evidence (GRADE)	Comments
Glucose (mmol/L)	MD 0.05 mmol/L lower with exercise (0.22 lower to 0.13 higher)	391 participants (45 studies)	⊕ ⊕ ⊕ ⊝ Moderate^a^	Glucose concentrations moderated by metabolic state, exercise mode, exercise duration and maximal aerobic capacity
Insulin (pmol/L)	MD 18.07 pmol/L lower with exercise (5.66 lower to 30.47 lower)	377 participants (38 studies)	⊕ ⊕ ⊕ ⊝ Moderate^b^	Insulin concentrations moderated by metabolic state
Glucagon (ng/L)	MD 24.60 ng/L higher with exercise (16.25 higher to 32.95 higher)	47 participants (5 studies)	⊕ ⊕ ⊕ ⊝ Moderate^c^	

*CI* confidence interval, *MD* mean difference

GRADE Working Group grades of evidence: High quality: we are very confident that the true effect lies close to that of the estimate of the effect. Moderate quality: we are moderately confident in the effect estimate: the true effect is likely to be close to the estimate of the effect, but there is a possibility that it is substantially different. Low quality: our confidence in the effect estimate is limited: the true effect may be substantially different from the estimate of the effect. Very low quality: we have very little confidence in the effect estimate: the true effect is likely to be substantially different from the estimate of effect

^a^There was considerable heterogeneity (*I*^2^ = 91.08%) that could not be explained by sub-group analyses or meta-regression

^b^There was considerable heterogeneity (*I*^2^ = 95.39%) that could not be explained by sub-group analyses or meta-regression

^c^There was considerable heterogeneity (*I*^2^ = 79.36%) that could not be explained by sub-group analyses or meta-regression

## Discussion

The aim of this review was to determine the effect of a single bout of continuous aerobic exercise on circulating glucose, insulin, and glucagon concentrations in healthy adults. Our results reveal that a single bout of aerobic exercise significantly decreases glucose and insulin concentrations when performed in the postprandial state, but not when performed in the fasted state. Glucose concentrations are decreased during cycle ergometer exercise but not treadmill exercise and changes in glucose concentrations are moderated by exercise duration (increased duration is associated with a smaller reduction) and participant $\dot{V}$O_2_ max (higher $\dot{V}$O_2_ max is associated with a smaller reduction). Our results also show that acute aerobic exercise significantly increases glucagon concentrations.

### The Effect of a Single Bout of Continuous Aerobic Exercise on Glucose Concentrations

Overall, acute aerobic exercise appeared to result in no meaningful change in glucose concentrations compared to resting conditions. However, when accounting for the metabolic state (postprandial or fasted), postprandial aerobic exercise resulted in a significant reduction in glucose concentrations. Acute aerobic exercise thus appears to be an effective method to reduce glucose concentrations in the postprandial state. This reduction is likely due to the induction of glucose transporter translocation and glucose transporter activity in skeletal muscle by exercise [69]. The upregulation of glucose transporter translocation and activity may not be secondary to insulin action, as exercise-stimulated glucose uptake has been demonstrated to occur independently of insulin [70], and glucose concentrations decreased in the context of decreasing insulin concentrations during postprandial aerobic exercise. Alternatively, exercise can increase insulin-dependent glucose uptake, possibly via a reduction in intramuscular glycogen and/or increase in AS160 phosphorylation [71]. The decrease in insulin concentrations (despite a reduction in glucose concentrations) may therefore reflect an increase in insulin sensitivity instead. Regardless of the mechanism responsible, this reduction is likely facilitated by the increase in microvascular recruitment and blood flow to skeletal muscle (thus increasing glucose delivery) caused by exercise [70]. The reduction in glucose concentrations is nevertheless small in magnitude and therefore the clinical significance of this finding is questionable. In contrast, no significant change in glucose concentrations during fasted aerobic exercise was detected. This is likely due to glucose concentrations already being low following an overnight fast [23], and that participants were individuals without diabetes, therefore making any further reduction difficult. The overall absence of a large decrease or increase in glucose concentrations does, however, highlight the high degree to which glucose concentrations are homeostatically regulated in non-diabetic populations.

The change in glucose concentrations during acute aerobic exercise was also influenced by exercise modality. Glucose concentrations were significantly reduced following acute aerobic exercise performed on a cycle ergometer, but non-significantly increased following aerobic exercise performed on a treadmill. Differences in glucose concentrations between exercise modalities have been reported previously [72, 73] and likely relate to underlying physiological differences between cycle ergometer and treadmill exercise [74], in particular the differences in carbohydrate metabolism between these exercise modalities [75]. Alternatively, this difference may be an artefact of the proportion of studies conducted in the postprandial and fasted state. Studies using a cycle ergometer were predominantly conducted in the postprandial state (~ 60% postprandial, ~ 40% fasted), whereas studies using a treadmill were predominantly conducted in the fasted state (~ 30% postprandial, ~ 70% fasted).

A higher participant $\dot{V}$O_2_ max and longer exercise duration were both associated with a smaller decrease in glucose concentrations. $\dot{V}$O_2_ max is positively correlated with insulin sensitivity [76], and therefore individuals that possess higher $\dot{V}$O_2_ max values are likely to have lower glucose concentrations in both the fed and fasted state, diminishing the extent to which glucose concentrations can be lowered by an intervention such as acute aerobic exercise. This also suggests that individuals possessing a lower level of cardiorespiratory fitness (and by inference a lower degree of insulin sensitivity) will see greater reductions in glucose concentrations with acute aerobic exercise. This is important considering individuals that possess a low level of cardiorespiratory fitness are at a greater risk of developing type 2 diabetes [77, 78]. The mechanism underlying the effect of exercise duration on glucose concentrations is unclear, but longer exercise durations may provide a larger window for homeostasis to be achieved following the initial disruption by aerobic exercise commencement. Nevertheless, the moderating effect of participant $\dot{V}$O_2_ max and exercise duration on glucose concentrations appears small when accounting for the magnitude of the overall effect of acute exercise on glucose concentrations.

### The Effect of a Single Bout of Continuous Aerobic Exercise on Insulin Concentrations

Acute aerobic exercise resulted in a significant reduction in circulating insulin concentrations relative to resting conditions. This reduction in insulin concentrations may partly reflect the decrease in glucose concentrations observed with postprandial aerobic exercise, which may be due to the stimulation of insulin-independent glucose uptake pathways in skeletal muscle by exercise [13]. Alternatively or additively, the reduction in insulin concentrations with exercise may be caused by an increase in insulin clearance [79], or an increase in insulin delivery (blood flow x blood insulin concentration) as a result of exercise-induced increases in skeletal muscle perfusion, decreasing insulin requirements and thus output [80]. When acute aerobic exercise is performed in the postprandial state, the effect of exercise on insulin concentrations is considerably greater. The present analysis therefore highlights that acute aerobic exercise is a potent tool for reducing postprandial insulin concentrations. In contrast, acute aerobic exercise undertaken in the fasted state resulted in a non-significant reduction in insulin concentrations. Short-term fasting (< 24 h) is well known to decrease insulin concentrations [23] and insulin levels would likely be at their lowest following > 6 h of fasting in non-diabetic individuals. Therefore, aerobic exercise performed in the fasted state is unlikely to prompt further reductions, especially when compared to fasted resting conditions. Changes in insulin concentrations were not significantly moderated by any covariate included in the meta-regression. This could be considered unexpected given that changes in glucose concentrations were moderated by participant $\dot{V}$O_2_ max and exercise duration. However, this finding is consistent with previous meta-regression analyses investigating the effect of acute exercise on the concentrations of other circulating hormones [81]. This meta-analysis reported no significant effect of sex, participant $\dot{V}$O_2_ max, exercise duration, or exercise intensity on the acyl-ghrelin or peptide YY response to acute exercise, suggesting that the hormonal milieu in response to acute exercise is comparable across individuals irrespective of key characteristics (e.g. age, sex, exercise intensity).

### The Effect of a Single Bout of Continuous Aerobic Exercise on Glucagon Concentrations

To our knowledge, this is the first review to quantify the changes in glucagon concentrations during exercise using a meta-analytical approach. The results from our analysis showed that acute aerobic exercise increased glucagon concentrations relative to resting conditions. Importantly, all five studies reported an increase in glucagon concentrations independent of metabolic state. This increase may be necessary to stimulate hepatic gluconeogenesis to provide substrate for contracting muscles and maintain euglycaemia [82], thus facilitating the absence of any large deviations in glucose concentrations. Despite the consistency of the glucagon response to acute aerobic exercise, the findings from this analysis should be treated with caution due to the small number of studies included. Future work should explore the effect of metabolic state (postprandial vs fasted) and exercise modality on changes in glucagon concentrations during acute aerobic exercise considering the limited number of studies currently available.

In addition to its glucoregulatory role, data from animal and human studies demonstrate that glucagon can decrease appetite [83] and therefore may be a key mechanism underlying exercise-induced anorexia [84]. However, glucagon concentrations are rarely measured during acute exercise studies, and consequently there are no reports of its association with appetite during or post-exercise. Future work investigating acute exercise and appetite should look to prioritize glucagon measurements (considering the consistent glucagon response to acute aerobic exercise) to evaluate the role of glucagon in exercise-induced anorexia.

### Limitations

There are several limitations to the present review and meta-analysis. Firstly, the application of these results is restricted to individuals who possess the same characteristics as those defined by the inclusion and exclusion criteria (healthy adults). The glucose, insulin and glucagon response to acute aerobic exercise in other patient populations cannot be assumed from our findings. Likewise, the results cannot be applied to other exercise modalities, such as high-intensity interval training or resistance training. The large heterogeneity observed in all three analyses is another limitation of the current review and meta-analysis but was expected considering the diversity in participant characteristics and experimental methodology used in acute exercise studies. Glucose, insulin and glucagon outcomes were consequently downgraded by one level using the GRADE approach and classified as moderate quality due to the large heterogeneity observed. Meal characteristics (e.g. timing and energy content) were not included in the meta-regression due to concerns regarding overfitting the model and therefore the influence of these properties on the study outcomes is unknown. The present review is also limited by the small number of studies measuring glucagon concentrations, which prevented sub-group meta-analyses and meta-regression from being performed, and thus the investigation of the influence of participant and intervention characteristics on this response. A further limitation is the use of pre- and post-exercise measurements to summarise the effect of acute exercise. While these measures represent the effect of acute exercise on glucose, insulin and glucagon concentrations at the point of exercise completion, they do not account for the temporal changes during exercise. Furthermore, these measures do not consider the effect of acute exercise on glucose, insulin and glucagon concentrations in the post-exercise period. Lastly, the majority of studies measuring glucose and/or insulin and/or glucagon concentrations were classified as having an unclear risk of bias overall. This was largely due to inadequate reporting of the randomisation process. Future investigations in this field should therefore report methodology in sufficient detail as described in the recent Proper Reporting of Evidence in Sport and Exercise Nutrition Trials (PRESENT) checklist proposed by Betts et al. [85].

## Conclusions

This systematic review and meta-analysis found that a single bout of continuous aerobic exercise had no significant effect on glucose concentrations, but significantly decreased insulin (~ 20 pmol/L) and significantly increased glucagon concentrations (~ 25 ng/L) relative to resting conditions in healthy adults. Sub-group analyses, however, revealed that the glucose and insulin responses were significantly moderated by metabolic state. A single bout of continuous aerobic exercise significantly decreased glucose (~ 0.3 mmol/L) and insulin (~ 40 pmol/L) concentrations when performed in the postprandial state (within 6 h of meal ingestion), but had no significant effect in the fasted state (at least 6 h after last meal ingestion) relative to resting conditions. Aerobic exercise undertaken in the postprandial state, therefore, appears to improve acute glycaemic control, and when considering that humans spend the majority of their waking time in this metabolic state, may be an important mechanism by which exercise activity reduces cardiometabolic disease risk.

## Supplementary Information

Below is the link to the electronic supplementary material.

Supplementary file1 (DOCX 17 kb)

Supplementary file2 (DOCX 27 kb)

Supplementary file3 (DOCX 18 kb)

Supplementary file4 (DOCX 18 kb)

Supplementary file5 (PDF 75 kb)

Supplementary file6 (PDF 125 kb)

Supplementary file7 (PDF 125 kb)

Supplementary file8 (PDF 303 kb)

Supplementary file9 (PDF 123 kb)

Supplementary file10 (PDF 123 kb)

## Declarations

### Availability of data and material

Please contact the corresponding author for data requests.
