# Supplementary material for: The Effect of a Single Bout of Continuous Aerobic Exercise on Glucose, Insulin and Glucagon Concentrations Compared to Resting Conditions in Healthy Adults: A Systematic Review, Meta-Analysis and Meta-Regression
Source: Sports Med. 2021 Apr 27;51(9):1949–66. doi: 10.1007/s40279-021-01473-2 (PMC8363558; doi:10.1007/s40279-021-01473-2)
Supplement: Supplementary file 1 — Supplementary file1 (DOCX 17 kb) [file 40279_2021_1473_MOESM1_ESM.docx]

**Title**

The effect of a single bout of continuous aerobic exercise on glucose, insulin and glucagon concentrations compared to resting conditions in non-diabetic adults: A systematic review, meta-analysis and meta-regression.

**Journal**

Sports Medicine

**Authors**

James Frampton^1, 2*^, Benjamin Cobbold^1^, Mikhail Nozdrin^1^, Htet T. H. Oo^1^, Holly Wilson^1^, Kevin G. Murphy^2^, Gary Frost^1^, Edward S. Chambers^1^

^1^Section for Nutrition Research, Department of Metabolism, Digestion and Reproduction, Faculty of Medicine, Imperial College London, London W12 0NN, United Kingdom.

^2^Section of Endocrinology and Investigative Medicine, Department of Metabolism, Digestion and Reproduction, Faculty of Medicine, Imperial College London, London W12 0NN, United Kingdom

***Corresponding Author**

James Frampton

Department of Metabolism, Digestion and Reproduction,

Faculty of Medicine,

Imperial College London,

London W12 0NN

United Kingdom

Email: j.frampton17@imperial.ac.uk

**DECLARATIONS**

**Funding**

No sources of financial assistance were used to conduct this study or to assist in the preparation of the manuscript. The Section of Endocrinology and Investigative Medicine is funded by grants from the MRC, BBSRC and NIHR, and is supported by the NIHR Biomedical Research Centre Funding Scheme. J.F is funded by the Imperial College London President’s PhD Scholarship.

**Conflicts of interest**

James Frampton, Benjamin Cobbold, Mikhail Nozdrin, Htet Oo, Holly Wilson, Kevin Murphy, Gary Frost and Edward Chambers declare that they have no conflicts of interest relevant to the content of this review.

**Availability of Data and Material**

Please contact the corresponding author for data requests.

**Author Contributions**

J.F and E.S.C conceived and designed the study. J.F performed databases searches. J.F, B.C, M.N, H.T.H.O and H.W participated in the screening process. J.F extracted data and performed all statistical analyses. J.F, E.S.C, K.G.M and G.F interpreted results of the analysis. J.F. wrote the initial draft of the manuscript, and critically revised by E.S.C, K.G.M and G.F. All authors read and approved the final manuscript.

**Electronic Supplementary Material Appendix S1**: Database search strategies

CENTRAL

*Searched:* 11/05/2020

"exercise OR run OR running OR cycle OR cycling OR walk OR walking in Record Title

AND ""glucose"" OR ""insulin"" OR ""glucagon"" in Title Abstract Keyword

AND rest OR resting OR control OR ctrl OR ""no exercise"" OR sedentary OR crossover OR cross-over OR counterbalanced in Abstract"

CINAHL

*Searched:* 10/04/2020

"TI (exercise or run or running or cycle or cycling or walk or walking)

AND AB (glucose or insulin or glucagon)

AND AB (rest or resting or control or ctrl or &quot;noexercise&quot; or sedentary or crossover or cross-over or counterbalanced)"

Embase

*Searched:* 10/04/2020

1. (exercise or run or running or cycle or cycling or walk or walking).ti.

2. (glucose or insulin or glucagon).ab,ti.

3. (rest or resting or control or ctrl or ""no exercise"" or sedentary or crossover or cross-over or counterbalanced).ab.

4. 1 and 2 and 3

Global Health

*Searched:* 10/04/2020

1. (exercise or run or running or cycle or cycling or walk or walking).ti.

2. (glucose or insulin or glucagon).ab,ti.

3. (rest or resting or control or ctrl or ""no exercise"" or sedentary or crossover or cross-over or counterbalanced).ab.

4. 1 and 2 and 3

HMIC

*Searched:* 10/04/2020

1. (exercise or run or running or cycle or cycling or walk or walking).ti.

2. (glucose or insulin or glucagon).ab,ti.

3. (rest or resting or control or ctrl or ""no exercise"" or sedentary or crossover or cross-over or counterbalanced).ab.

4. 1 and 2 and 3

Medline

*Searched:* 10/04/2020

1. (exercise or run or running or cycle or cycling or walk or walking).ti.

2. (glucose or insulin or glucagon).ab,ti.

3. (rest or resting or control or ctrl or ""no exercise"" or sedentary or crossover or cross-over or counterbalanced).ab.

4. 1 and 2 and 3

PubMed

*Searched:* 31/03/2020

"(((exercise[Title] OR run[Title] OR running[Title] OR cycle[Title] OR cycling[Title] OR walk[Title] OR walking[Title]))

AND (glucose[Title/Abstract] OR insulin[Title/Abstract] OR glucagon[Title/Abstract]))

AND (rest[Title/Abstract] OR resting[Title/Abstract] OR control[Title/Abstract] OR ctrl[Title/Abstract] OR ""no exercise""[Title/Abstract] OR sedentary[Title/Abstract] OR crossover[Title/Abstract] OR cross-over[Title/Abstract] OR counterbalanced[Title/Abstract]) "

PscyInfo

*Searched:* 10/04/2020

1. (exercise or run or running or cycle or cycling or walk or walking).ti.

2. (glucose or insulin or glucagon).ab,ti.

3. (rest or resting or control or ctrl or ""no exercise"" or sedentary or crossover or cross-over or counterbalanced).ab.

4. 1 and 2 and 3

ScienceDirect

*Searched:* 11/04/2020

Title: exercise OR run OR running OR cycle OR cycling OR walk OR walking

Title, abstract, keywords: glucose OR insulin OR glucagon

Terms: rest OR resting OR control OR ctrl OR ""no exercise"" OR sedentary OR crossover OR cross-over OR counterbalanced"

Scopus

*Searched:*07/04/2020

(TITLE(exercise OR run OR running OR cycle OR cycling OR walk OR walking)

AND TITLE-ABS-KEY(glucose OR insulin OR glucagon)

AND TITLE-ABS-KEY(rest OR resting OR control OR ctrl OR ""no exercise"" OR sedentary OR crossover OR cross-over OR counterbalanced))

Web of Science

*Searched:* 10/04/2020

"TITLE: (exercise OR run OR running OR cycle OR cycling OR walk OR walking)

AND TOPIC: (glucose OR insulin OR glucagon)

AND TOPIC: (rest OR resting OR control OR ctrl OR ""no exercise"" OR sedentary OR crossover OR cross-over OR counterbalanced)"
