# Supplementary material for: The Effect of a Single Bout of Continuous Aerobic Exercise on Glucose, Insulin and Glucagon Concentrations Compared to Resting Conditions in Healthy Adults: A Systematic Review, Meta-Analysis and Meta-Regression
Source: Sports Med. 2021 Apr 27;51(9):1949–66. doi: 10.1007/s40279-021-01473-2 (PMC8363558; doi:10.1007/s40279-021-01473-2)
Supplement: Supplementary file 6 — Supplementary file6 (PDF 125 kb) [file 40279_2021_1473_MOESM6_ESM.pdf]

Title

The effect of a single bout of continuous aerobic exercise on glucose, insulin and glucagon concentrations compared to resting conditions in non-diabetic adults: A systematic review, meta-analysis and meta-regression.

Journal

Sports Medicine

Authors

James Frampton[1, 2*], Benjamin Cobbold[1], Mikhail Nozdrin[1], Htet T. H. Oo[1], Holly Wilson[1], Kevin G. Murphy[2], Gary Frost[1], Edward S. Chambers[1]

[1]Section for Nutrition Research, Department of Metabolism, Digestion and Reproduction, Faculty of Medicine, Imperial College London, London W12 0NN, United Kingdom.

[2]Section of Endocrinology and Investigative Medicine, Department of Metabolism, Digestion and Reproduction, Faculty of Medicine, Imperial College London, London W12 0NN, United Kingdom

***Corresponding Author**

James Frampton

Department of Metabolism, Digestion and Reproduction,

Faculty of Medicine,

Imperial College London,

London W12 0NN

United Kingdom

Email: j.frampton17@imperial.ac.uk

Electronic Supplementary Material Figure S2

DECLARATIONS

Funding

No sources of financial assistance were used to conduct this study or to assist in the preparation of the manuscript. The Section of Endocrinology and Investigative Medicine is funded by grants from the MRC, BBSRC and NIHR, and is supported by the NIHR Biomedical Research Centre Funding Scheme. J.F is funded by the Imperial College London President's PhD Scholarship.

Conflicts of interest

James Frampton, Benjamin Cobbold, Mikhail Nozdrin, Htet Oo, Holly Wilson, Kevin Murphy, Gary Frost and Edward Chambers declare that they have no conflicts of interest relevant to the content of this review.

Availability of Data and Material

Please contact the corresponding author for data requests.

Author Contributions

J.F and E.S.C conceived and designed the study. J.F performed databases searches. J.F, B.C, M.N, H.T.H.O and H.W participated in the screening process. J.F extracted data and performed all statistical analyses. J.F, E.S.C, K.G.M and G.F interpreted results of the analysis. J.F. wrote the initial draft of the manuscript, and critically revised by E.S.C, K.G.M and G.F. All authors read and approved the final manuscript.

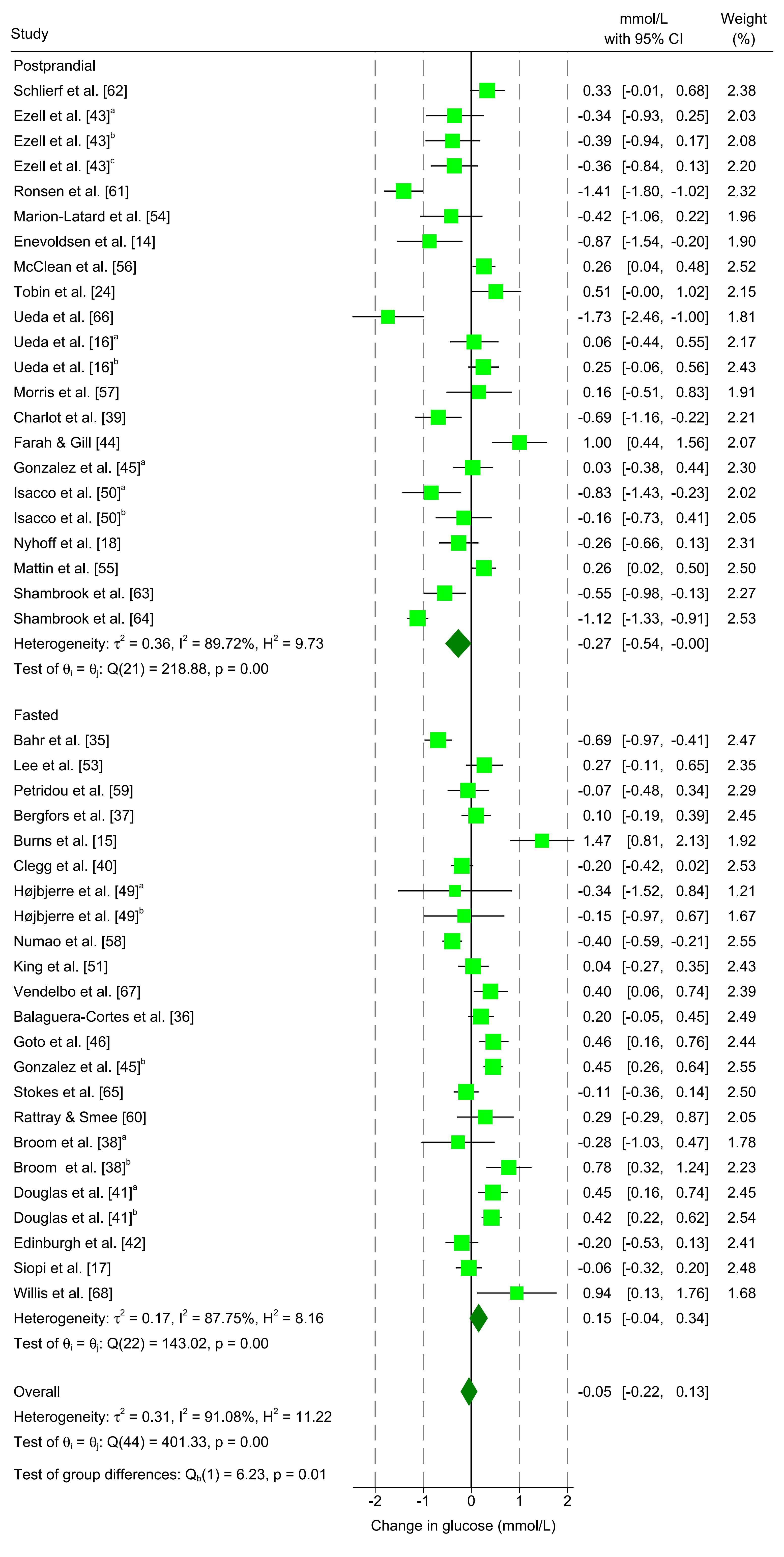
Study
mmol/L with 95% CI
Weight (%)
Postprandial
Schlierf et al. [62] 0.33 [-0.01, 0.68] 2.38
Ezell et al. [43][a] -0.34 [-0.93, 0.25] 2.03
Ezell et al. [43][b] -0.39 [-0.94, 0.17] 2.08
Ezell et al. [43][c] -0.36 [-0.84, 0.13] 2.20
Ronsen et al. [61] -1.41 [-1.80, -1.02] 2.32
Marion-Latard et al. [54] -0.42 [-1.06, 0.22] 1.96
Enevoldsen et al. [14] -0.87 [-1.54, -0.20] 1.90
McClean et al. [56] 0.26 [0.04, 0.48] 2.52
Tobin et al. [24] 0.51 [-0.00, 1.02] 2.15
Ueda et al. [66] -1.73 [-2.46, -1.00] 1.81
Ueda et al. [16][a] 0.06 [-0.44, 0.55] 2.17
Ueda et al. [16][b] 0.25 [-0.06, 0.56] 2.43
Morris et al. [57] 0.16 [-0.51, 0.83] 1.91
Charlot et al. [39] -0.69 [-1.16, -0.22] 2.21
Farah & Gill [44] 1.00 [0.44, 1.56] 2.07
Gonzalez et al. [45][a] 0.03 [-0.38, 0.44] 2.30
Isacco et al. [50][a] -0.83 [-1.43, -0.23] 2.02
Isacco et al. [50][b] -0.16 [-0.73, 0.41] 2.05
Nyhoff et al. [18] -0.26 [-0.66, 0.13] 2.31
Mattin et al. [55] 0.26 [0.02, 0.50] 2.50
Shambrook et al. [63] -0.55 [-0.98, -0.13] 2.27
Shambrook et al. [64] -1.12 [-1.33, -0.91] 2.53
Heterogeneity: $\tau^2 = 0.36$, $I^2 = 89.72\%$, $H^2 = 9.73$ -0.27 [-0.54, -0.00]
Test of $\theta_i = \theta_j$: Q(21) = 218.88, p = 0.00
Fasted
Bahr et al. [35] -0.69 [-0.97, -0.41] 2.47
Lee et al. [53] 0.27 [-0.11, 0.65] 2.35
Petridou et al. [59] -0.07 [-0.48, 0.34] 2.29
Bergfors et al. [37] 0.10 [-0.19, 0.39] 2.45
Burns et al. [15] 1.47 [0.81, 2.13] 1.92
Clegg et al. [40] -0.20 [-0.42, 0.02] 2.53
Højbjerre et al. [49][a] -0.34 [-1.52, 0.84] 1.21
Højbjerre et al. [49][b] -0.15 [-0.97, 0.67] 1.67
Numao et al. [58] -0.40 [-0.59, -0.21] 2.55
King et al. [51] 0.04 [-0.27, 0.35] 2.43
Vendelbo et al. [67] 0.40 [0.06, 0.74] 2.39
Balaguera-Cortes et al. [36] 0.20 [-0.05, 0.45] 2.49
Goto et al. [46] 0.46 [0.16, 0.76] 2.44
Gonzalez et al. [45][b] 0.45 [0.26, 0.64] 2.55
Stokes et al. [65] -0.11 [-0.36, 0.14] 2.50
Rattray & Smee [60] 0.29 [-0.29, 0.87] 2.05
Broom et al. [38][a] -0.28 [-1.03, 0.47] 1.78
Broom et al. [38][b] 0.78 [0.32, 1.24] 2.23
Douglas et al. [41][a] 0.45 [0.16, 0.74] 2.45
Douglas et al. [41][b] 0.42 [0.22, 0.62] 2.54
Edinburgh et al. [42] -0.20 [-0.53, 0.13] 2.41
Siopi et al. [17] -0.06 [-0.32, 0.20] 2.48
Willis et al. [68] 0.94 [0.13, 1.76] 1.68
Heterogeneity: $\tau^2 = 0.17$, $I^2 = 87.75\%$, $H^2 = 8.16$ 0.15 [-0.04, 0.34]
Test of $\theta_i = \theta_j$: Q(22) = 143.02, p = 0.00
Overall -0.05 [-0.22, 0.13]
Heterogeneity: $\tau^2 = 0.31$, $I^2 = 91.08\%$, $H^2 = 11.22$
Test of $\theta_i = \theta_j$: Q(44) = 401.33, p = 0.00
Test of group differences: $Q_b(1) = 6.23$, p = 0.01
-2
-1
0
1
2
Change in glucose (mmol/L)
