# Supplementary material for: The Effect of a Single Bout of Continuous Aerobic Exercise on Glucose, Insulin and Glucagon Concentrations Compared to Resting Conditions in Healthy Adults: A Systematic Review, Meta-Analysis and Meta-Regression
Source: Sports Med. 2021 Apr 27;51(9):1949–66. doi: 10.1007/s40279-021-01473-2 (PMC8363558; doi:10.1007/s40279-021-01473-2)
Supplement: Supplementary file 10 — Supplementary file10 (PDF 123 kb) [file 40279_2021_1473_MOESM10_ESM.pdf]

Title

The effect of a single bout of continuous aerobic exercise on glucose, insulin and glucagon concentrations compared to resting conditions in non-diabetic adults: A systematic review, meta-analysis and meta-regression.

Journal

Sports Medicine

Authors

James Frampton[1, 2*], Benjamin Cobbold[1], Mikhail Nozdrin[1], Htet T. H. Oo[1], Holly Wilson[1], Kevin G. Murphy[2], Gary Frost[1], Edward S. Chambers[1]

[1]Section for Nutrition Research, Department of Metabolism, Digestion and Reproduction, Faculty of Medicine, Imperial College London, London W12 0NN, United Kingdom.

[2]Section of Endocrinology and Investigative Medicine, Department of Metabolism, Digestion and Reproduction, Faculty of Medicine, Imperial College London, London W12 0NN, United Kingdom

***Corresponding Author**

James Frampton

Department of Metabolism, Digestion and Reproduction,

Faculty of Medicine,

Imperial College London,

London W12 0NN

United Kingdom

Email: j.frampton17@imperial.ac.uk

Electronic Supplementary Material Figure S6

DECLARATIONS

Funding

No sources of financial assistance were used to conduct this study or to assist in the preparation of the manuscript. The Section of Endocrinology and Investigative Medicine is funded by grants from the MRC, BBSRC and NIHR, and is supported by the NIHR Biomedical Research Centre Funding Scheme. J.F is funded by the Imperial College London President's PhD Scholarship.

Conflicts of interest

James Frampton, Benjamin Cobbold, Mikhail Nozdrin, Htet Oo, Holly Wilson, Kevin Murphy, Gary Frost and Edward Chambers declare that they have no conflicts of interest relevant to the content of this review.

Availability of Data and Material

Please contact the corresponding author for data requests.

Author Contributions

J.F and E.S.C conceived and designed the study. J.F performed databases searches. J.F, B.C, M.N, H.T.H.O and H.W participated in the screening process. J.F extracted data and performed all statistical analyses. J.F, E.S.C, K.G.M and G.F interpreted results of the analysis. J.F. wrote the initial draft of the manuscript, and critically revised by E.S.C, K.G.M and G.F. All authors read and approved the final manuscript.

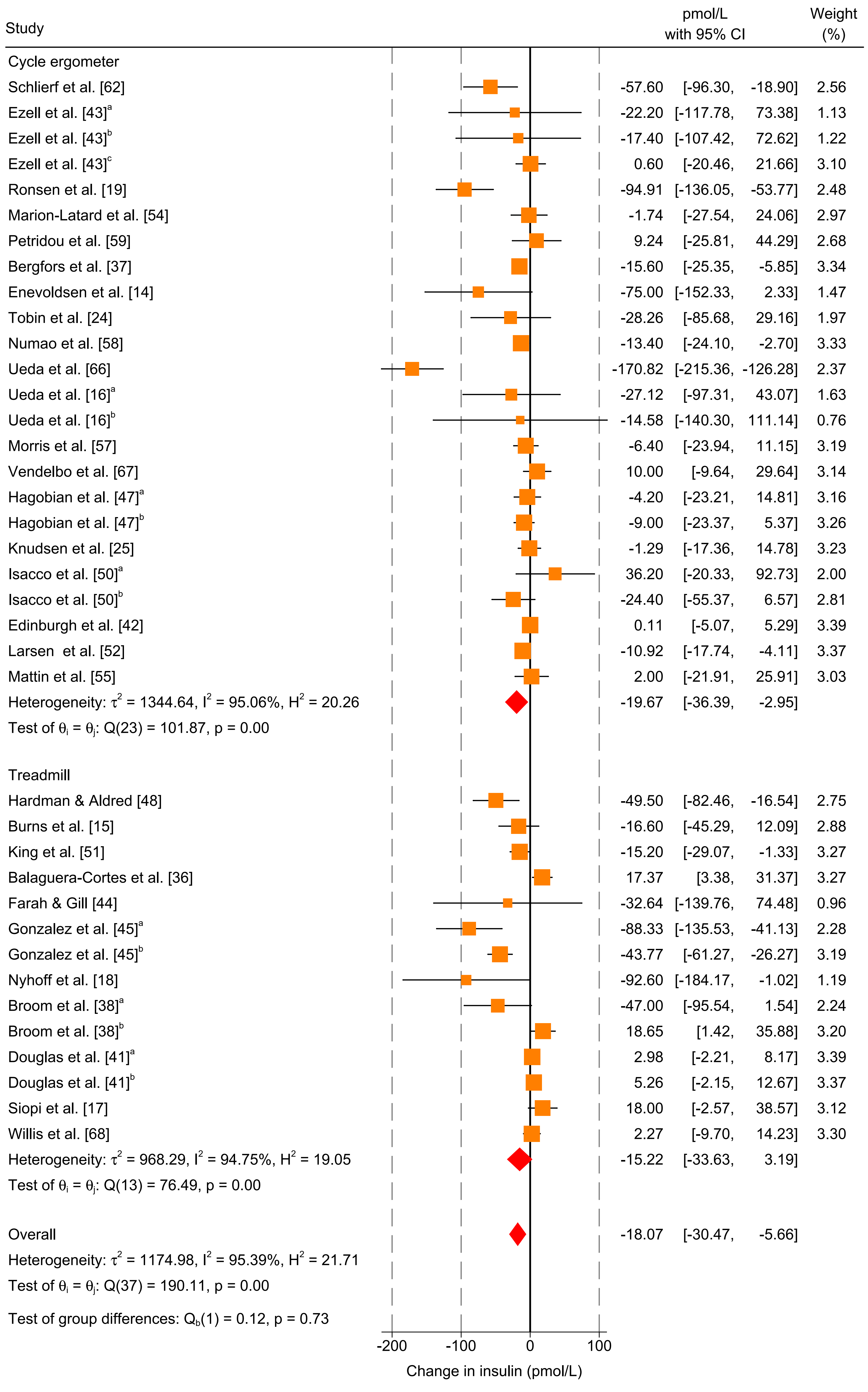

Study
pmol/L with 95% CI
Weight (%)
Cycle ergometer
Schlierf et al. [62] -57.60 [-96.30, -18.90] 2.56
Ezell et al. [43][a] -22.20 [-117.78, 73.38] 1.13
Ezell et al. [43][b] -17.40 [-107.42, 72.62] 1.22
Ezell et al. [43][c] 0.60 [-20.46, 21.66] 3.10
Ronsen et al. [19] -94.91 [-136.05, -53.77] 2.48
Marion-Latard et al. [54] -1.74 [-27.54, 24.06] 2.97
Petridou et al. [59] 9.24 [-25.81, 44.29] 2.68
Bergfors et al. [37] -15.60 [-25.35, -5.85] 3.34
Enevoldsen et al. [14] -75.00 [-152.33, 2.33] 1.47
Tobin et al. [24] -28.26 [-85.68, 29.16] 1.97
Numao et al. [58] -13.40 [-24.10, -2.70] 3.33
Ueda et al. [66] -170.82 [-215.36, -126.28] 2.37
Ueda et al. [16][a] -27.12 [-97.31, 43.07] 1.63
Ueda et al. [16][b] -14.58 [-140.30, 111.14] 0.76
Morris et al. [57] -6.40 [-23.94, 11.15] 3.19
Vendelbo et al. [67] 10.00 [-9.64, 29.64] 3.14
Hagobian et al. [47][a] -4.20 [-23.21, 14.81] 3.16
Hagobian et al. [47][b] -9.00 [-23.37, 5.37] 3.26
Knudsen et al. [25] -1.29 [-17.36, 14.78] 3.23
Isacco et al. [50][a] 36.20 [-20.33, 92.73] 2.00
Isacco et al. [50][b] -24.40 [-55.37, 6.57] 2.81
Edinburgh et al. [42] 0.11 [-5.07, 5.29] 3.39
Larsen et al. [52] -10.92 [-17.74, -4.11] 3.37
Mattin et al. [55] 2.00 [-21.91, 25.91] 3.03
Heterogeneity: τ^2 = 1344.64, I^2 = 95.06%, H^2 = 20.26 -19.67 [-36.39, -2.95]
Test of $\theta_i = \theta_j$: Q(23) = 101.87, p = 0.00
Treadmill
Hardman & Aldred [48] -49.50 [-82.46, -16.54] 2.75
Burns et al. [15] -16.60 [-45.29, 12.09] 2.88
King et al. [51] -15.20 [-29.07, -1.33] 3.27
Balaguera-Cortes et al. [36] 17.37 [3.38, 31.37] 3.27
Farah & Gill [44] -32.64 [-139.76, 74.48] 0.96
Gonzalez et al. [45][a] -88.33 [-135.53, -41.13] 2.28
Gonzalez et al. [45][b] -43.77 [-61.27, -26.27] 3.19
Nyhoff et al. [18] -92.60 [-184.17, -1.02] 1.19
Broom et al. [38][a] -47.00 [-95.54, 1.54] 2.24
Broom et al. [38][b] 18.65 [1.42, 35.88] 3.20
Douglas et al. [41][a] 2.98 [-2.21, 8.17] 3.39
Douglas et al. [41][b] 5.26 [-2.15, 12.67] 3.37
Siopi et al. [17] 18.00 [-2.57, 38.57] 3.12
Willis et al. [68] 2.27 [-9.70, 14.23] 3.30
Heterogeneity: τ^2 = 968.29, I^2 = 94.75%, H^2 = 19.05 -15.22 [-33.63, 3.19]
Test of $\theta_i = \theta_j$: Q(13) = 76.49, p = 0.00
Overall -18.07 [-30.47, -5.66]
Heterogeneity: τ^2 = 1174.98, I^2 = 95.39%, H^2 = 21.71
Test of $\theta_i = \theta_j$: Q(37) = 190.11, p = 0.00
Test of group differences: $Q_b(1)$ = 0.12, p = 0.73
-200
-100
0
100
Change in insulin (pmol/L)
